# Blocking interleukin-4 enhances efficacy of vaccines for treatment of opioid abuse and prevention of opioid overdose

**DOI:** 10.1038/s41598-018-23777-6

**Published:** 2018-04-03

**Authors:** Megan Laudenbach, Federico Baruffaldi, Christine Robinson, Philipp Carter, Davis Seelig, Carly Baehr, Marco Pravetoni

**Affiliations:** 1 Minneapolis Medical Research Foundation, Minneapolis, MN USA; 2 Department of Veterinary Clinical Sciences, University of Minnesota College of Veterinary Medicine, St. Paul, MN USA; 3 Department of Veterinary Population Medicine, University of Minnesota College of Veterinary Medicine, St. Paul, MN USA; 4 Department of Medicine and Pharmacology, Center for Immunology, University of Minnesota Medical School, Minneapolis, MN USA

## Abstract

Vaccines offer an option to treat heroin and prescription opioid abuse and prevent fatal overdoses. Opioid vaccines elicit antibodies that block opioid distribution to the brain and reduce opioid-induced behavioral effects and toxicity. The major limitation to the translation of addiction vaccines is that efficacy is observed only in subjects achieving optimal drug-specific serum antibody levels. This study tested whether efficacy of a vaccine against oxycodone is increased by immunomodulators targeting key cytokine signaling pathways involved in B and T cell lymphocyte activation. Blockage of IL-4 signaling increased vaccine efficacy in blocking oxycodone distribution to the brain and protection against opioid-induced behavior and toxicity in mice. This strategy generalized to a peptide-protein conjugate immunogen, and a tetanus-diphtheria-pertussis vaccine. These data demonstrate that cytokine-based immunomodulators increase efficacy of vaccines against small molecules, peptides and proteins, and identify IL-4 as a pharmacological target for improving efficacy of next-generation vaccines.

## Introduction

Vaccines are the single most effective intervention ever devised in medical practice^1^. Vaccines have the potential to protect against a variety of communicable and non-communicable diseases, including substance use disorders associated with abuse of stimulants and opioids. Worldwide, over 33 million people abuse drugs, whose health consequences include blood-borne pathogen transmission, cancer, and death^2^. In the US, 2.6 million people are dependent on heroin and prescription opioids including oxycodone and hydrocodone, costing more than $95 billion in crime, lost work productivity and health care annually^3^. Over 50,000 opioid-related fatal overdoses occur each year, making opioid abuse the leading cause of accidental death in the US, and prompting recent increases in stringency of prescription guidelines^3^. These data suggest that current interventions are insufficient to curb opioid abuse, and that more therapeutic options are needed^4^.

Vaccines and other biologics against substance use disorders have shown pre-clinical and clinical proof of concept, but no product has reached the market^5–10^. A major limitation to the translation of addiction vaccines is that clinical efficacy is observed only in the fraction of immunized subjects that display high levels of drug-specific antibodies^5,11^. This is a common shortcoming of vaccines consisting of purified protein subunits, or of proteins conjugated to peptides, carbohydrates, or synthetic small molecule haptens. For instance, the increased incidence in whooping cough is partially attributable to switching from the whole-cell pertussis vaccine to formulations containing the purified acellular pertussis subunit^12^. To increase efficacy, synthetic vaccines against substance use disorders are mixed with adjuvants or delivered via particle platforms^5^. However, a limited number of adjuvants or delivery systems are approved for human use because of potential side effects, and available adjuvants trigger innate immunity, but do not directly stimulate adaptive immunity^13,14^, suggesting that more effective immunization strategies are needed.

Screening of bioconjugates with varying hapten design, conjugation chemistry, carrier choice, adjuvant and immunization regimen identified a candidate vaccine consisting of an oxycodone-based hapten conjugated to keyhole limpet hemocyanin (KLH) through a tetraglycine linker (OXY-KLH)^15–19^. The OXY-KLH vaccine generates antibodies that selectively bind oxycodone and hydrocodone in serum, block their distribution to the brain, and prevent their behavioral effects, including oxycodone intravenous self-administration^17^. The OXY-KLH vaccine is also effective in preventing oxycodone-induced respiratory depression, and it does not interfere with naloxone reversal of opioid toxicity in rats^20^. The pre-clinical efficacy profile of OXY-KLH supports its translation, but also warrants its use as a model to test novel strategies to improve efficacy of vaccines against substance use disorders.

After immunization, generation of antibodies (Ab) results from T cell-dependent B cell activation and germinal center (GC) formation in secondary lymphoid organs^21,22^. Within the GC, specialized CD4^+^ T follicular helper (Tfh) cells help cognate antigen-specific B cells differentiate into long-lived switched immunoglobulin memory cells and antibody-secreting B cells^21,22^. We found that frequency of naïve and early antigen-specific B or T cells correlates with individual vaccine efficacy against nicotine or opioids^23–25^. Greater vaccine efficacy also correlated with increased GC activation^23^. These data suggest that vaccine efficacy can be enhanced by strategies that modulate activation and differentiation of vaccine-specific B and T cell populations. The canonical Th_1_ signature cytokines IL-2 and IL-7 inhibit GC formation by limiting Tfh differentiation^26,27^, whereas IL-4 drives Th_2_ responses and suppresses IgG subclass shift from IgG_1_ to IgG_2a_^28^. Immunization success or failure hinges on the fine balance between these cytokines and other co-stimulatory factors, and their downstream effects on B and T cells.

This study tested interleukins as a pharmacological target to enhance vaccine efficacy against drugs of abuse and other targets. Immune checkpoint inhibitors, interleukins, and monoclonal antibodies (mAb) against interleukins or interleukin receptors have been shown to increase efficacy of CD8^+^ T cell-mediated immunotherapies^29,30^. Combining a HIV gag/pol vaccine with IL-4 receptor antagonism induced both HIV-specific CD8^+^ T cells, and gag-specific B cell immunity paired to enhancement of IgG_2a_ class switching in mice^31^. However, the effect of immunomodulators on subunit or conjugate vaccines that rely on CD4^+^ T cell-dependent B cell activation to generate antibodies remains largely unexplored. Since small molecules and biologic immunomodulators of IL-4, IL-2 and IL-7 are either approved or close to FDA approval for different indications, this study focused on testing the novel hypothesis that modulation of IL-4, IL-2 and IL-7 enhanced efficacy of the OXY-KLH vaccine against oxycodone. An αIL-4 mAb (Pascolizumab®) has been tested for allergies and pulmonary tuberculosis (NCT01638520), and other therapeutics targeting IL-4 (e.g., Altrakincept®), IL-4Rα (e.g., Dupilumab®, AMG-317, and Pitrakinra®) are under development for treatment of atopic dermatitis, allergies, autoimmune disorders, or other indications^32^. An αCD25 mAb (anti-IL-2αR, Daclizumab®) is approved for prevention of kidney transplant rejection, treatment of melanoma and other cancers^33^. An αCD127 mAb (anti-IL-2αR) is being evaluated for treatment of autoimmune disorders (NCT02293161) and multiple sclerosis (NCT01808482). Repurposing of approved or commercially available immunomodulators can accelerate translation of this approach and development of clinically viable vaccine formulations.

In this study, we found that IL-4 depletion enhanced OXY-KLH’s efficacy against oxycodone and increased the subset of vaccine responders. The increased efficacy correlated with higher IgG_2a_ titers and a mixed Th_1_/Th_2_ response. This strategy generalized to a peptide-protein conjugate immunogen and a vaccine for infectious diseases. Our data demonstrate that targeting of IL-4 is a safe and effective strategy to improve efficacy of vaccines against substance use disorders and other unmet medical needs.

## Results

### Vaccine efficacy is enhanced by modulators of IL-4 signaling

First, this study tested whether modulation of either Th_2_ signaling through IL-4 or Th_1_ signaling through IL-2 improved efficacy of the OXY-KLH vaccine. In sequential rounds of screening, independent mouse cohorts were immunized with lead vaccine formulations and challenged with increasing doses of oxycodone (2.25–10 mg/kg). These drug challenges model clinically relevant oxycodone doses and resulting plasma concentrations found in recreational opioid users and individuals in pain management regimens^34–40^. As predetermined screening criteria, new leads had to be more effective than OXY-KLH in aluminum adjuvant to be tested in subsequent rounds. We predicted that blocking signaling through IL-2 or IL-7, or promoting IL-4 signaling, would increase efficacy through increased GC formation. In the first round, OXY-KLH was administered in combination with immunomodulators including recombinant IL-4 (rIL-4), rIL-4+ recombinant IL-21 (rIL-21), or with antibodies against the IL-2 receptor (αCD25). Immunocomplexes of interleukins and anti-interleukin mAb (IL:αIL) are potentially more effective than interleukins alone^41^ and have longer half-life^42,43^. For example, IL-2:αIL-2 and IL-7:αIL-7 potentiate cellular immunity against cancer by enhancing activation of the IL-2 or IL-7 receptor α chain (IL-2αR or IL-7αR)^44^, whereas immunocomplexes consisting of IL-4 and a neutralizing mAb against IL-4 (αIL-4, clone 11B11) showed increased activity at the IL-4α receptor and protection against parasites^45^. Thus, we also tested whether αIL-4 or the IL-4:αIL-4 complex enhanced vaccine efficacy.

**Figure 1:**
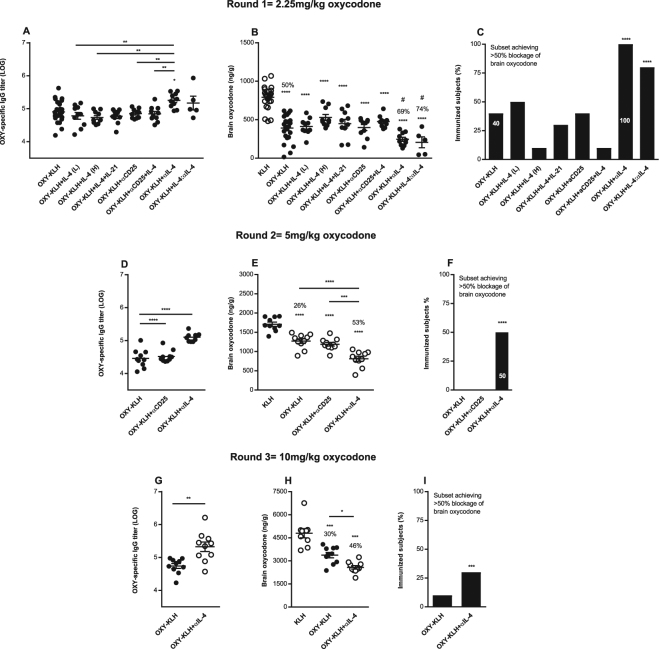
Modulation of IL-4 signaling enhances anti-oxycodone antibody, vaccine efficacy against oxycodone, and the subset of immunized subjects that showed vaccine efficacy. Three independent cohorts of male BALB/c mice were immunized s.c. with OXY-KLH and KLH adsorbed on alum adjuvant and challenged a week after the 3^rd^ immunization with increasing doses of oxycodone (2.25–10 mg/kg s.c.). Interleukins were administered at days 0, 14 and 28 in combination with each immunization. The mAb was administered 2 days prior and 1 day after the 1^st^ immunization. Experimental groups: (**A**–**C**) KLH (n = 24), OXY-KLH (n = 25), and OXY-KLH plus: rIL-4 (30,000 IU, s.c.), rIL-4 (60,000 IU, s.c.), IL-4 plus IL-21 (60,000 IU total, s.c.), αIL-2 alpha receptor mAb (αCD25, 1.0 mg, i.p., n = 10), αCD25 mAb (1.0 mg, i.p.) plus IL-4 (30,000 IU, s.c., n = 10), αIL-4 mAb (1.0 mg, i.p., n = 10), or IL-4:αIL-4 mAb (30,000 IU and 0.5 mg of mAb mixed prior to injection, s.c., n = 5), and challenged with 2.25 mg/kg oxycodone. (**D–F**) KLH, OXY-KLH, and OXY-KLH plus αCD25 mAb (1.0 mg, i.p.), or αIL-4 mAb (1.0 mg, i.p.) and challenged with 5 mg/kg oxycodone (n = 10/group). (**G**–**I**) KLH, OXY-KLH, and OXY-KLH plus αIL-4 mAb (1.0 mg, i.p.) and challenged with 10 mg/kg oxycodone (n = 10/group). Shown oxycodone-specific IgG titers, oxycodone distribution to the brain, and fraction (%) of immunized subjects that showed more than 50% reduction in oxycodone distribution to the brain compared to the mean brain concentration in the KLH control group. Data are mean ± SEM. Data shown are from at least two independent experiments. Titers and oxycodone concentrations analyzed by one-way ANOVA and Tukey’s multiple comparisons test or unpaired two-tailed t-test. Chi-square test: (**C**) (χ^2^ = 282.8, df = 8), F) (χ^2^ = 120.0, df = 2), and I) (χ^2^ = 12.5, df = 1). Percentages (%) above bars indicate reduction compared to KLH. *p < 0.05, **p < 0.001, ****p < 0.0001 compared to KLH, and brackets or # indicate significance between groups, ^#^p < 0.05.

Surprisingly, co-administration of OXY-KLH and rIL-4 did not increase Ab titers or vaccine efficacy (Fig. 1A–C), and co-administration of OXY-KLH with both IL-4 and IL-21 did not increase vaccine efficacy. Contrary to our expectations, blocking IL-2 signaling with the αCD25 mAb or co-administration of αCD25 with IL-4 did not enhance OXY-KLH efficacy. Instead, both the αIL-4 mAb and the rIL-4:αIL-4 immunocomplex showed significantly increased efficacy compared to OXY-KLH alone in blocking a dose of 2.25 mg/kg oxycodone (Fig. 1B,C). Finally, co-administration of OXY-KLH with the αCD127 mAb targeting the IL-7 receptor (Suppl. Fig. S1), or with the co-stimulatory molecules programmable death ligand 1 or ICOS ligand (Suppl. Fig. S2) did not increase vaccine efficacy.

Because FDA guidelines favor clinical testing of individual components of biologics, it will be a simpler pathway to seek regulatory approval for an αIL-4 mAb targeting endogenous IL-4 instead of the pre-mixed rIL-4:αIL-4 immunocomplex. Thus, in the subsequent rounds of screening, mice were vaccinated with the OXY-KLH plus αIL-4 and further challenged with oxycodone at 5 mg/kg (Fig. 1D–F) or 10 mg/kg (Fig. 1G–I). In both rounds, αIL-4 increased oxycodone-specific serum IgG titers, and the subset of immunized mice that responded to the vaccine. The effect of αIL-4 on OXY-KLH efficacy was not due to immunogenicity of the rat anti-mouse mAb, because the rat anti-mouse αCD25 (Fig. 1D–F) or the rat anti-mouse αCD127 (Suppl. Fig. S1) did not alter vaccine immunogenicity and efficacy. From this screening, we identified OXY-KLH plus αIL-4 mAb as our lead formulation, and we then proceeded to further characterize its efficacy, safety, and applicability to other vaccine platforms.

### Combining OXY-KLH plus αIL-4 mAb is effective across immunization regimens

**Figure 2:**
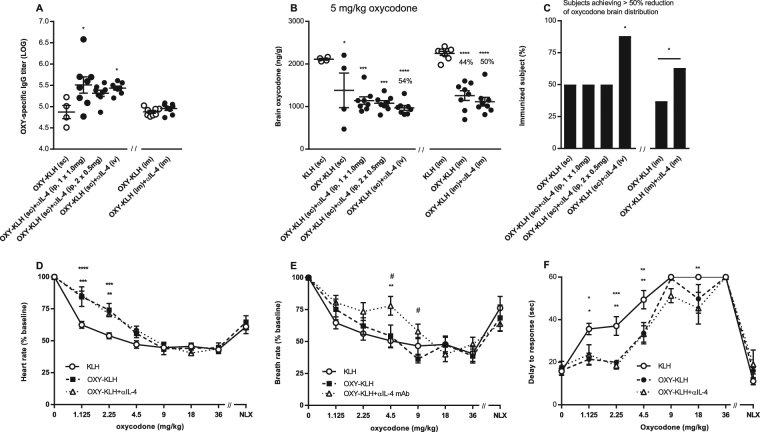
The lead formulation of OXY-KLH plus αIL-4 mAb is effective across clinically-relevant immunization regimens and attenuated oxycodone-induced physiological and behavioral effects. BALB/c mice were immunized with OXY-KLH or KLH on days 0, 14 and 28, and tested for vaccine efficacy a week after the third immunization. Mice received KLH or OXY-KLH s.c. or i.m., whereas αIL-4 mAb was administered i.p., i.v., or i.m. In a first cohort, mice were challenged with 5 mg/kg oxycodone: (**A**) oxycodone-specific IgG titers, (**B**) oxycodone brain concentrations, and (**C**) subjects that showed more than 50% reduction in oxycodone distribution to the brain compared to the KLH group. Data are from two independent experiments and expressed as mean ± SEM. Percentages (%) above bars indicate reduction compared to KLH. One-way ANOVA paired with Tukey’s multiple comparisons test or unpairedtwo-tailed t-test. Chi-square test (χ^2^ = 62.66, df = 6). *p < 0.05, **p < 0.001, ****p < 0.0001 compared to KLH, and brackets to indicate significance between groups. In a second cohort, immunized mice were tested for opioid-induced respiratory depression, heart rate and antinociception. Oxycodone was administered s.c. every 15 min at increasing doses and the doses listed are the cumulative dose received. Naloxone 0.1 mg/kg s.c. was administered 15 min after the final oxycodone dose to reverse opioid effects. (**D**) heart rate, (**E**) breath rate, and (**F**) antinociception on a hot plate. Data are mean ± SEM. Data shown are from one experiment (n = 8). Two-way ANOVA paired with Tukey’s multiple comparisons test. *p < 0.05, **p < 0.01, ***p < 0.001 compared to KLH. ^#^p < 0.05, ^##^p < 0.01 for OXY-KLH compared to OXY-KLH plus anti-IL-4 mAb.

To ensure ease of translation, the formulation of OXY-KLH and αIL-4 was tested across clinically relevant routes of administration and found to be effective (Fig. 2A–C and Suppl. Fig. S3). Importantly, αIL-4 enhanced the efficacy of OXY-KLH when both agents were administered intramuscularly.

### Combining OXY-KLH and αIL-4 mAb blunted oxycodone-induced behavioral and toxic effects

To further explore the clinical relevance of this approach, we tested whether immunization blocks oxycodone-induced respiratory depression and bradycardia, which are common side effects contributing to overdose-related death and complications^46^. Immunized mice were also tested for oxycodone analgesia on a hotplate as a measure of vaccine efficacy in blocking opioid central effects^15,16^. OXY-KLH was effective in preventing oxycodone-induced depression of heart (Fig. 2D) and respiratory (Fig. 2E) rates as well as blocking oxycodone-induced analgesia over increasing doses (Fig. 2F). Co-administration of OXY-KLH and αIL-4 mAb potentiated the effect of the vaccine on respiratory depression evoked by a cumulative dose of 4.5 mg/kg oxycodone. Additionally, the efficacy of naloxone in reversing opioid-induced analgesia, bradycardia, and respiratory depression was preserved in both OXY-KLH and OXY-KLH plus αIL-4 mAb groups compare to KLH control. These data support that opioid vaccines can be combined with naloxone to rescue from fatal overdose.

### Blockage of IL-4 shifts IgG subclass distribution

**Figure 3:**
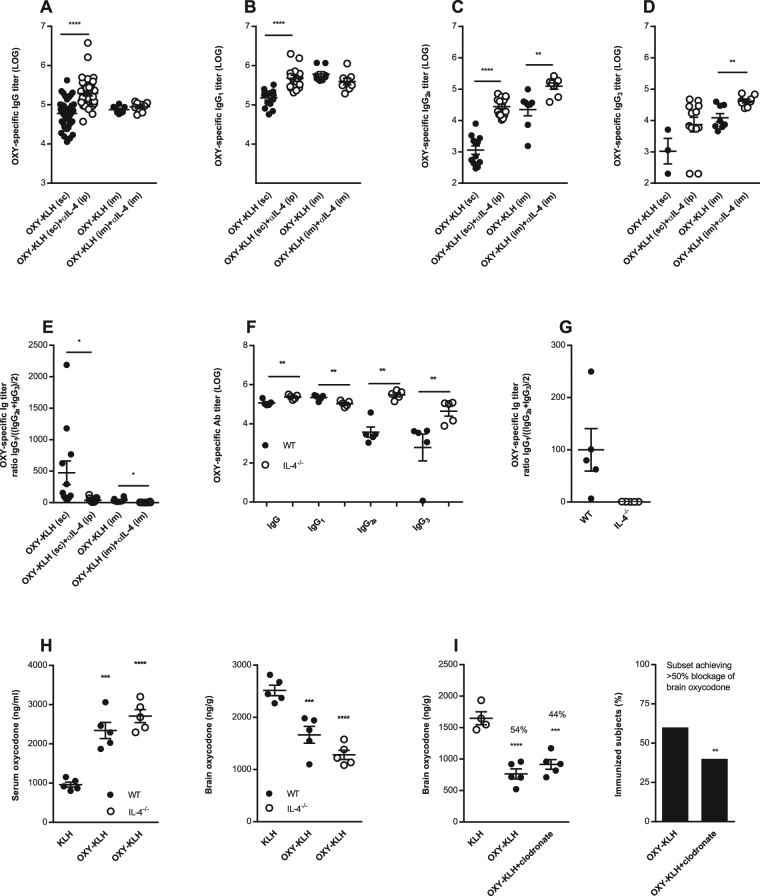
Depletion and ablation of IL-4 shifts post-vaccination IgG subclass distribution. (**A**–**E**) Serum anti-oxycodone IgG antibodies were analyzed for IgG_1_, IgG_2a_, and IgG_3_ subclass in mice immunized with OXY-KLH ± αIL-4 mAb from experiments shown in Figs 1A–H and 2A–C. (**A**) oxycodone-specific IgG titers in mice immunized with OXY-KLH s.c., n = 49; OXY-KLH s.c. + IL-4 mAb i.p., n = 38; OXY-KLH i.m., n = 8; OXY-KLH i.m. + IL-4 mAb i.m., n = 8). IgG subclass analysis was performed on a randomly selected sample from each immunization group (n = 5–6 mice/group): (**B**) IgG_1_, (**C**) IgG_2a_, (**D**) IgG_3_, (**E**) IgG_1_/((IgG_2a_ + IgG_3_)/2). (**F**–**H**) In a separate study, IL-4^−/−^ and wild-type mice controls were immunized with KLH or OXY-KLH in alum (s.c., n = 5 each group). (**F**) oxycodone-specific IgG titers by subclass, (**G**) IgG_1_/((IgG_2a_ + IgG_3_)/2), and (**H**) effect of immunization on oxycodone distribution to serum and brain. (**I**) In a follow-up independent experiment, mice were immunized with either KLH (s.c., n = 4) or OXY-KLH (s.c., n = 10). The OXY-KLH group received either saline or liposome-encapsulated clodronate to deplete macrophages (n = 5), and 24 hrs later challenged with 5.0 mg/kg oxycodone. Data are from one experiment. (**I**) vaccine efficacy in blocking oxycodone distribution to the brain (left) and the subset (%) of immunized mice showing efficacy (right). Data are mean ± SEM. (**A**–**G**) unpaired two-tailed t-test. (**H**,**I**) One-way ANOVA paired with Tukey’s test, and chi-square test (χ^2^ = 8.00, df = 1, p = 0.0047). *p < 0.05, **p < 0.01, ***p < 0.001, ****p < 0.0001.

To understand the mechanism underlying the effect of IL-4 depletion on vaccine efficacy, we examined the distribution of IgG subclasses in immunized BALB/c mice. Regardless of the route of administration, co-administration of OXY-KLH and αIL-4 induced oxycodone-specific serum IgG_2a_ and IgG_3_ antibodies (Fig. 3A–D) and decreased the ratio of IgG_1_/((IgG_2a_ + IgG_3_)/2) (Fig. 3E). Consistent with our previous reports, increased oxycodone-specific serum IgG antibody titers correlated with increased OXY-KLH efficacy (Suppl. Fig. S4). Furthermore, increased oxycodone-specific serum IgG_1_ and IgG_2a_ titers also correlated to vaccine efficacy against oxycodone (Suppl. Fig. S4). The effect of αIL-4 on IgG_2a_ generalized across genetic backgrounds, since combining OXY-KLH and αIL-4 also increased IgG_2a_ titers in C57Bl/6 mice (Suppl. Fig. S5). These results are consistent with previous reports that αIL-4 increased IgG_2a_ responses in mice immunized with a penicillin-based hapten conjugated to tetanus toxoid (TT)^47,48^. These data suggest a role for drug-specific serum IgG_2a_ antibodies in mediating efficacy of vaccines for substance use disorders.

To gain insight in the possible mechanism underlying the effect of the αIL-4 mAb on vaccine efficacy, we next immunized IL-4-deficient (IL-4^−/−^) mice with OXY-KLH. IL-4^−/−^ mice showed increased OXY-KLH efficacy, which was associated with increased production of IgG_2a_ and IgG_3_ (Fig. 3F–H), in agreement with previous reports that indicate that immunization or infection of IL-4 KO mice elicit increased IgG_2a_ antibodies^49^. Similarly, when OXY-KLH was administered in combination with an αIL-4 mAb that is non-neutralizing^42^, vaccine efficacy was not affected (Suppl. Fig. S6). Together, these results show that the effect of the neutralizing mAb, clone 11B11, is due to a reduction in IL-4 signaling.

**Figure 4:**
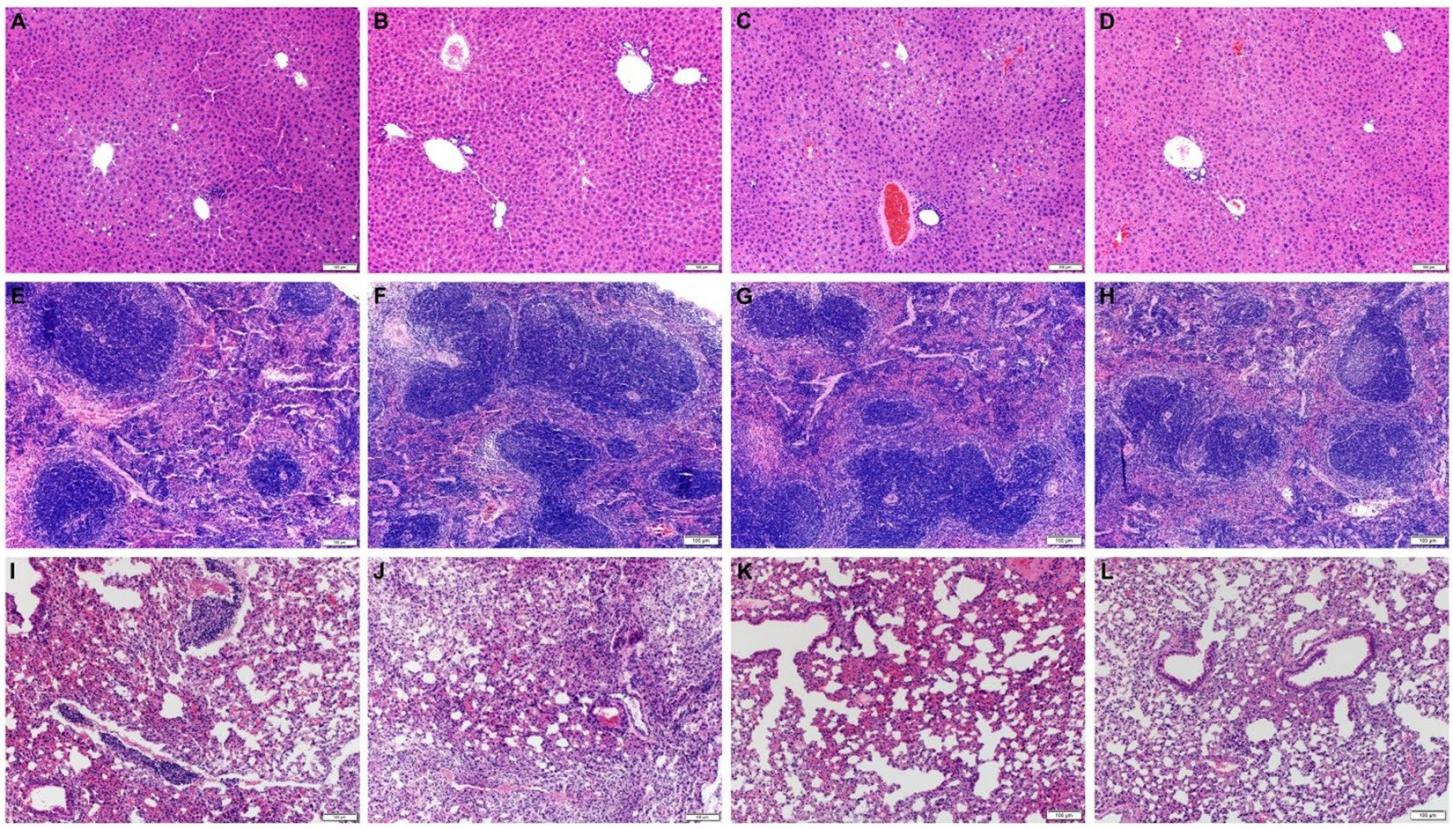
No histopathologic differences were identified in BALB/c mice treated with KLH, OXY-KLH, αIL-4 mAb, and OXY-KLH plus αIL-4 mAb (n = 3/group). (**A**–**D**) liver, (**E**–**H**) spleen and (**I**–**L**) lungs. Other organs shown in Suppl. Fig. S7.

In both IL-4 depleted mice and IL-4^−/−^ mice, greater vaccine efficacy against oxycodone was associated with increased IgG_2a_ and IgG_3_ antibody titers. This finding is consistent with other reports that active immunization against drugs of abuse benefits from a mixed Th_1_/Th_2_ response^50–54^, but its biological relevance remains unknown. We hypothesized that IgG-dependent effector functions may be involved at least in part in vaccine efficacy against drugs of abuse. IgG_2a_ and IgG_3_ activate various effector functions, including phagocytosis through the Fcγ receptors I-IV^55^. To test the hypothesis that antibody-mediated phagocytosis is required for vaccine efficacy against drugs of abuse, mice immunized with OXY-KLH were administered liposome-embedded clodronate, an *in vivo* macrophage poison^56^, and then challenged with 5 mg/kg oxycodone. Treatment with clodronate reduced the efficacy of OXY-KLH in preventing oxycodone distribution to the brain (Fig. 4I). This is the first report of IgG-dependent phagocytosis as a potential mechanism underlying efficacy of addiction vaccines. Future studies will further explore the role of IgG subclasses in mediating the effect of polyclonal and monoclonal antibodies against opioids and other drugs of abuse.

### Co-administration of OXY-KLH and αIL-4 mAb is potentially safe

All mice that received the combination of OXY-KLH s.c. and αIL-4 i.p. were evaluated for potential adverse effects. Mice receiving this formulation exhibited no significant difference in baseline nociception or body weight as compared to mice that received KLH control (Suppl. Table S1). An independent cohort of BALB/c mice immunized with KLH, OXY-KLH, αIL-4, or OXY-KLH plus αIL-4 underwent a clinical toxicology (Suppl. Table S2) and histopathology assessment (Fig. 4 and Suppl. Fig. S7) to evaluate the safety profile of this approach. Across groups, serum protein, bilirubin, urea nitrogen, and cholesterol concentrations as well as alanine aminotransferase (ALT) activity were within the reference interval. In all groups, elevations in aspartate aminotransferase (AST) activity compared to reference values were noted and, in light of the unremarkable ALT activity and the liver histopathology, is not likely the result of mAb treatment. The histopathology of treated animals revealed mild to occasionally moderate vacuolar change within the centrolobular hepatocytes that was similar in severity across all treatment groups with no significant pathology in the spleen. However, lungs from all groups displayed mild to occasionally moderately increased numbers of interstitial inflammatory cells, principally lymphocytes with fewer neutrophils with concurrent congestion. Occasionally, these regions of inflammation were centered on lymphocyte-rich lymphatic vessels. The severity of the lung pathology was similar across the four treatment groups. No significant pathology was identified in other organs (Suppl. Fig. S7).

### IL-4 modulation does not interfere with post-immunization hapten-specific B cell early expansion, but increases peptide-specific T cell differentiation

**Figure 5:**
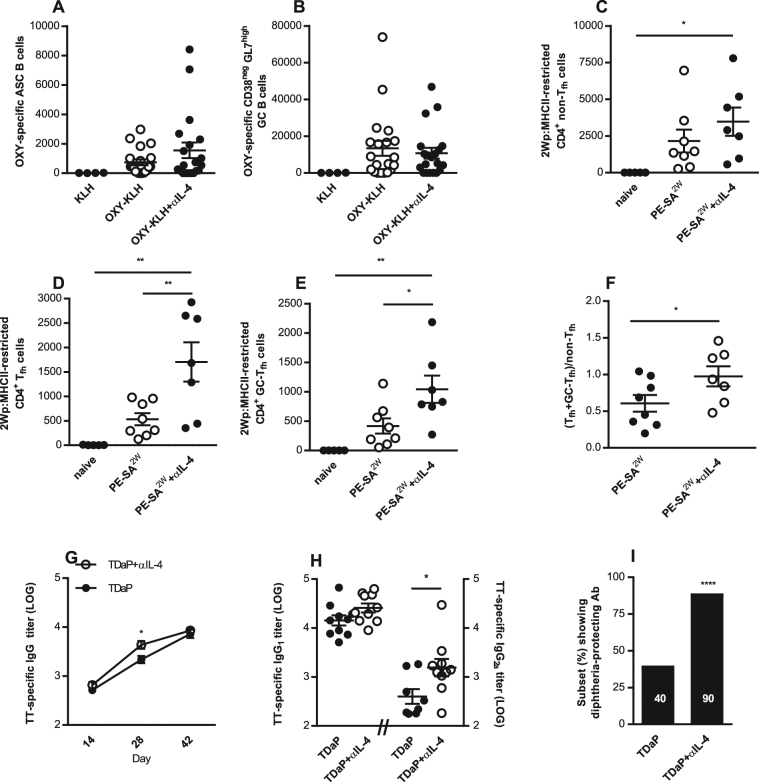
IL-4 depletion does not impair post-immunization hapten-specific B cell early expansion, increases peptide-specific T cell differentiation, and enhances efficacy of a tetanus-diphtheria-pertussis vaccine. (**A**,**B**) BALB/c mice immunized once with KLH (s.c., n = 4), OXY-KLH (s.c., n = 20), and OXY-KLH (s.c.) plus αIL-4 mAb (i.p, n = 20). At 7 days after immunization, analysis of OXY-specific B cells in pooled lymph nodes and spleen by flow cytometry: (**A**) Antibody secreting cells, and (**B**) GC B cells. Data shown are from three experiments. (**C**–**F**) C57Bl/6 naïve mice (n = 5) and mice immunized once with PE-SA^2W^ (n = 8) and PE-SA^2W^ plus αIL-4 mAb (n = 8). At 7 days after immunization, analysis of 2W-specific CD4^+^ T cells in pooled lymph nodes and spleen by flow cytometry: (**C**) non-Tfh cells, (**D**) Tfh cells, (**E**) GC-Tfh cells, (**F**) (Tfh + GC- Tfh)/non-Tfh. Data shown are from two experiments. (**G**–**I**) Mice immunized with TDaP and TDaP plus αIL-4 mAb (n = 10 each group). (**G**) TT-specific IgG titer over time, (**H**) TT-specific IgG_1_ and IgG_2a_ titers on day 28 after the first immunization, and (**I**) subset (%) of subjects showing anti-diphtheria protective antibodies. Data shown are from one experiment. Data are mean ± SEM. (**A**–**E**) One-way ANOVA paired with Tukey’s multiple comparisons test. (**F** and **H**) unpaired two tailed t-test. (**G**) Two-way ANOVA paired with Tukey’s multiple comparisons test. (**I**) Chi-square (χ^2^ = 52.43, df = 1). *p < 0.05, **p < 0.01 versus control, or brackets to indicate specific group differences.

Since co-administration of OXY-KLH and αIL-4 increased vaccine efficacy against oxycodone, we sought to gain a better understanding of the effects of IL-4 depletion on GC *in vivo* in our model. In mice immunized with OXY-KLH, depletion of IL-4 did not affect early expansion of OXY-specific antibody-secreting and GC B cell subsets (Fig. 5A,B, and Suppl. Fig. S8 for analysis detail). These data suggest that hapten-specific B cells responses were at least in part IL-4-independent at this early stage.

The formation and persistence of GC requires functional and anatomical synergy between GC B cells and GC- Tfh^57^. Here we tested the effect of IL-4 depletion on T cell responses to immunization with a model immunogen, 2W peptide conjugated to streptavidin-phycoerythrin (SA-PE). The ^2W^SA-PE immunogen allows tracking of 2W-specific T cells by 2Wp:MHCII tetramer technology paired to flow cytometry to assess T cell differentiation, and models peptide–protein vaccines. Co-administration of ^2W^SA-PE plus αIL-4 increased Tfh differentiation toward GC- Tfh, the critical T cell helper subset devoted to sustain cognate GC B cell expansion (Fig. 5C–F, and Suppl. Fig. S9-10 for analysis detail). At 7 days after immunization, αIL-4 increased antigen-specific non-Tfh expansion (Fig. 5C), which may explain IgG subclass shift toward IgG_2a_ and IgG_3_ observed in mice immunized with OXY-KLH plus αIL-4 (Fig. 3). Most importantly, αIL-4 shifted T cell differentiation toward Tfh and GC- Tfh (Fig. 5F). These data support the use of mAb against IL-4 to increase efficacy of vaccines consisting of carrier proteins conjugated to peptide antigens, such as cancer-derived or Alzheimer’s β amyloid-derived peptides.

### IL-4 modulation increases efficacy of tetanus-diphtheria-pertussis vaccine

Current acellular pertussis vaccines are safer but not more effective than whole-cell pertussis vaccines. Here we found that co-administration of the αIL-4 mAb with TDaP (GSK©) improved TT-specific serum IgG titers over time (Fig. 5G), and increased TT-specific IgG_2a_ titers (Fig. 5H). In addition, an *in vitro* assay was used to evaluate Ab-mediated protection of Vero cells from diphtheria toxin. Co-administration of αIL-4 with TDaP significantly increased the subset of immunized subjects that produced antibodies that protected cells from the toxin (Fig. 5I and Fig. S10). These data suggest that depletion of IL-4 is a strategy that can be used to enhance efficacy of vaccines for multiple applications.

## Discussion

This study tested the novel hypothesis that the efficacy of vaccines for substance use disorders is enhanced by immunomodulators that target key interleukin signaling pathways involved in critical B and T cell functions underlying antibody production. Here, we found that combining a candidate vaccine against the prescription opioid oxycodone with an IL-4 neutralizing mAb enhanced vaccine efficacy in blocking oxycodone distribution to the brain, and increased the subset of immunized subjects that displayed vaccine efficacy. Both the OXY-KLH vaccine and the combination of OXY-KLH with αIL-4 mAb were effective in blocking oxycodone-induced behavioral effects and preventing respiratory depression and bradycardia in mice challenged with increasing doses of oxycodone. The effect of OXY-KLH on preventing oxycodone-induced adverse effects that contribute to overdose death supported findings from another report that oxycodone vaccines prevented oxycodone overdose mortality^58^. In this study, both the OXY-KLH vaccine and the combination of OXY-KLH with αIL-4 mAb did not interfere with reversal by naloxone of respiratory depression and bradycardia. These data replicated and extended evidence that OXY-KLH did not interfere with naloxone and extended-release naltrexone in mice and rats^20^, supporting the notion that opioid vaccines can be safely combined with current pharmacotherapy against opioid abuse and overdose. The combination of OXY-KLH with αIL-4 mAb was effective across multiple routes of administration, and no significant adverse effects were observed, indicating that modulation of IL-4 is a potentially safe method that can be used to enhance vaccine efficacy against a variety of targets or in at-risk populations.

Co-administration of the OXY-KLH vaccine plus αIL-4 mAb or immunization of IL-4^−/−^ mice yielded a more balanced Th_1_/Th_2_ response as demonstrated by the presence of IgG_1_ as well as IgG_2a_ and IgG_3_ in contrast to a primarily Th_2_ (i.e., IgG_1_ only) response in mice immunized with OXY-KLH in alum. While other groups have shown that more effective formulations of addiction vaccines elicit a more balanced Th_1_/Th_2_ response^50–53^, the biological relevance of IgG_2a_ and IgG_3_ in the context of addiction vaccines is still unknown. Our data support the hypothesis that blockage of IL-4 induced a shift toward IgG_2a_ and IgG_3_, and that presence of IgG_2a_ increased vaccine efficacy against oxycodone. For instance, IgG_2a_ is a key mediator for efficacy of influenza vaccines, and it is possible that IgG_2a_ also elicits antibody-dependent effector functions that increase removal or inactivation of drugs of abuse. In this study, ablation of macrophages using clodronate *in vivo* reduced OXY-KLH vaccine efficacy against oxycodone, indicating a possible role for antibody-mediated phagocytosis of opioids. Future studies will focus on addressing the contribution of antibody-dependent effector functions to efficacy of vaccines and mAb against opioid abuse and overdose.

This study suggested that IL-4 signaling and specific IgG subclasses mediate efficacy of addiction vaccines in mice. We previously reported that the frequency of naïve and early antigen-specific B and T cell subsets correlated with individual vaccine efficacy against nicotine or opioids in mice^23–25^. Greater GC formation was also associated with increased vaccine efficacy against oxycodone in mice^23^. In addition to offering insight into the immunobiology of addiction vaccines, these data provide benchmarks for pre-clinical and clinical development of addiction vaccines. The frequency of antigen-specific B and T cells in human blood paired with analysis of IL-4 genetic polymorphisms and drug-specific IgG subclasses may also provide biomarkers predictive of vaccine efficacy in human subjects.

Combining immunomodulators with active immunization was also effective at increasing GC-Tfh differentiation in response to a model peptide-protein conjugate vaccine, as well as increasing the efficacy of the TDaP vaccine. This suggests the potential for broader application of immunomodulators to enhance efficacy of subunit or conjugate vaccines that may benefit from a mixed Th_1_/Th_2_-type response or from a specific IgG subclass distribution. This strategy may generalize to sensitive populations including pregnant women, elderly, or immunocompromised individuals known to be poor responders to vaccines. This study provided additional pre-clinical proof of efficacy that opioid vaccines offer a viable approach to the treatment of opioid abuse and prevention of opioid fatal overdoses.

## Methods

### Ethics statement

Studies were approved by the Minneapolis Medical Research Foundation and the University of Minnesota Institutional Animal Care and Use Committees. All procedures were performed in accordance with the Guide for the Care and Use of Laboratory Animals (8th Edition, National Academies Press).

### Drugs and immunomodulators

Mouse rIL-4 and rIL-21 were obtained from Bio-Techne Corp., Minneapolis, MN. Monoclonal Ab αIL-4 (rat anti-mouse IgG_1_, clone 11B11), αCD25 (rat anti-mouse IgG_1_, clone 7D4) and αCD127 (rat anti-mouse IgG_2a_, clone A7R34) were obtained from Bio X Cell, West Lebanon, NH.

### Vaccines and immunogens

The oxycodone-based hapten was synthesized from oxycodone HCl, and included a tetraglycine linker^16^. The conjugation was performed in 0.1 M MES buffer pH 5.5 using a final EDAC concentration of 208 mM, and purified with Amicon filters with a cut-off of 100KDa, (Amicon, EMD Millipore Corp., Billerica, MA). The model immunogen phycoerythryin (PE) conjugated to streptavidin (SA) (ProZyme, Inc., Hayward, CA) was conjugated to the biotinylated 2W peptide (PE-SA^2W^). Conjugation of PE-SA^2W^ was performed at a molar ratio of 4.5:1 in 0.01 M PBS for 30 minutes at a final concentration of 1 mg/ml, and purified by filtration (Amicon, 100KDa) and centrifugation at 4000 × *g* for 15 minutes at 4 °C. Tetanus toxoid, diphtheria, and acellular pertussis (TDaP) vaccine (BOOSTRIX, GlaxoSmithKline plc, Isleworth, London, England) was obtained from the Hennepin County Medical Center pharmacy.

### Animals

Male BALB/c and C57BL/6 mice were obtained from Harlan Laboratories, Madison, WI. IL-4 deficient (IL-4^−/−^) mice and wild-type controls were obtained from The Jackson Laboratory, Bar Harbor, ME (stock no. 002496 and 000651, respectively). Mice were housed with a 12-h light/12-h dark cycle and fed *ad libitum*.

### Immunization regimen

BALB/c, C57BL/6, or IL-4^−/−^ mice were immunized with 75 µg OXY-KLH or unconjugated KLH at days 0, 14 and 28. The OXY-KLH and the unconjugated KLH were adsorbed on an equal volume of alum adjuvant (Alhydrogel ‘85’, 2%, Brenntag Biosector, Denmark) prior to administration either s.c. or i.m. as noted in each figure. Recombinant interleukin 4 (30,000 IU and 60,000 IU) and rIL-4 plus rIL-21 (60,000 IU total) were administered s.c. at days 0, 14 and 28 in combination with each immunization. The rIL-4:αIL-4 mAb immunocomplex was mixed prior to injection (30,000 IU and 0.5 mg of mAb) and administered once s.c. on day 0. Monoclonal antibodies were administered (0.5 mg/mouse/injection) two days prior and one day after the first immunization. The αIL-4 mAb was administered either i.p., i.v., or i.m. as noted in each figure. Analysis of the OXY-specific B cell repertoire was performed a week after the first immunization, whereas Ab characterization and oxycodone challenges were initiated a week after the third immunization. An independent cohort of BALB/c mice immunized with KLH or OXY-KLH was first analyzed for anti-oxycodone Ab titers, given liposome-embedded clodronate (Liposoma, Amsterdam, The Netherlands) to poison macrophages *in vivo*^56^, and then challenged with oxycodone. In some experiments, C57Bl/6 mice were immunized s.c. with 25 µg of the immunogen PE-SA^2W^ adsorbed on alum adjuvant, and analysis of the 2W-specific T cell repertoire was performed a week after the first immunization. In an independent study, male BALB/c mice were immunized s.c. on days 0, 14 and 28 with 25 µl of TDaP diluted in 0.01 M phosphate-buffered saline to a final volume of 100 µl, and given either saline or αIL-4 mAb i.p. two days prior and one day after the first immunization.

### Antibody analysis

Standard 96-well ELISA plates were coated with unconjugated chicken ovalbumin (OVA) or OXY hapten conjugated to OVA, at 5 ng/well in carbonate buffer at pH 9.6 and blocked with 1% gelatin^23^. Similarly, plates were coated with 50 ng/well of TT or OVA. Primary antibodies were incubated with goat-anti-mouse IgG, IgG_1_, IgG_2a_, or IgG_3_ conjugated to horseradish peroxidase to measure titers of oxycodone- or TT-specific IgG and individual IgG subclasses (Alpha Diagnostic International, Inc., San Antonio, TX).

### Analysis of antigen-specific B and T cells

Lymph nodes and/or spleen were collected at seven days after the first immunization. Using a two-step procedure, OXY-specific B cells were first isolated by magnetic enrichment using a Quadromacs Separator (Miltenyi Biotec, Auburn, CA) and biotinylated oxycodone-based haptens conjugated to PE-SA or a decoy reagent, anti-PE mAbs conjugated to magnetic particles (Miltenyi Biotec) and then analyzed by multi-parameter flow cytometry^23,25^ (and Suppl. Fig. S8). Similarly, antigen-specific T cells were first isolated by magnetic enrichment using 2W conjugated to soluble MHCII receptors (2Wp:MHCII) tetramerized on SA-PE and then analyzed by flow cytometry using standard surface and intracellular markers^23,59^. 2Wp:MHCII-restricted Tfh and GC- Tfh were further characterized as described^60^ (and Suppl. Fig. S9). Analysis was conducted on a LSRFortessa (BD Biosciences, San Jose, CA), and analyzed using FlowJo (FlowJo LLC, Ashland, OR).

### Vaccine efficacy against oxycodone distribution to the brain, oxycodone-induced behavioral and toxic effects

Independent cohorts of mice immunized with either KLH, OXY-KLH, or OXY-KLH in combination with immunomodulators were challenged with increasing doses of oxycodone (2.25–10 mg/kg, s.c.). At 30 min after the oxycodone challenge, mice were euthanized and oxycodone serum and brain concentrations measured by gas chromatography as described^23^. In a separate study, immunized mice were tested for vaccine efficacy in blocking opioid-induced respiratory depression, heart rate, and anti-nociception. Oxycodone-induced analgesia was first assessed on a hotplate as described^23^, and then arterial oxygen saturation (SaO_2_), breath rate, and heart rate were measured with a MouseOx oxymeter (STARR Life Sciences Corp., Oakmont, PA). Oxycodone was administered s.c. every 15 min at increasing doses and the doses listed are the cumulative dose received. The opioid antagonist naloxone 0.1 mg/kg s.c. was administered 15 min after the final oxycodone dose to reverse opioid effects.

### Clinical pathology

Mice were immunized s.c. with either KLH or OXY-KLH and given either saline or αIL-4 mAb i.p. as described above. A week after the third immunization, BALB/c mice were euthanized with CO_2_ to perform histopathology and clinical pathology. Serum was collected by centrifugation of clotted whole blood at 7500 rpm for three minutes at 4 °C and collected in lithium heparin (VACUTAINER, Becton, Dickinson and Co., Franklin Lakes, NJ). Frozen samples were sent to Marshfield Labs (Marshfield, WI) for analysis of protein, bilirubin, alanine aminotransferase (ALT), urea nitrogen, total cholesterol, and aspartate aminotransferase (AST). Organs were harvested, fixed in 10% neutral buffered formalin (methanol free, ultrapure EM grade, Polysciences, Inc., Warminster, PA), and embedded in paraffin using standard methods. Thereafter, four-micron tissue sections were cut and stained with hematoxylin and eosin (H&E). Histopathologic evaluation was performed by a board certified veterinary pathologist (DMS) who was blinded to their treatment status.

### *In vitro* diphtheria toxin protection assay

Mouse serum samples were first diluted 1:10 in RPMI containing 10% FBS and then serially diluted in 384-well plates using Bio-Tek Precision 2000 liquid-handlers (Bio-Tek, Winooski, VT). Forty µL of 100 ng/mL Diphtheria toxin (List Biological Laboratories, Campbell, CA) was added to each sample well and incubated for 1 h at 37 °C. Twenty µL of Vero cells (in Phenol Red-free DMEM containing 10% FBS [fDMEM], at a density 2 × 10^5^ cells/mL) were then added to each well and incubated 24 h at 37 °C. Following incubation, cells were washed using a plate washer (Bio-Tek, Winooski, VT), and 40 µL of fDMEM containing 0.15 mM resazurin was added to each well and incubated 16 hrs. Plates were read on a Spectramax M2 (Molecular Devices, Sunnyvale, CA) using ratiometric reads at 530/620 nm to evaluate cell viability. The IC_50_ for Vero cell survival was calculated, and the subset of immunized subjects that generated protective titers was defined as the proportion of samples in each group with IC_50_ >10.

### Data Analysis

Mean titers, number of B and T cells, oxycodone concentrations, breath rate, hearth rate, and delay time to nociception across groups were analyzed by one- or two-way ANOVA followed by Tukey’s multiple comparisons test. Unpaired two-tailed t-tests were used to analyze data between two groups. Chi-square or Fisher’s test were used to analyze the subset (%) of immunized mice that showed vaccine efficacy against oxycodone or diphtheria-protecting titers.

### Data availability

Supplementary information is available in the online version of the paper. Source data used to generate all figures or any other information are available from the corresponding author upon request.

## Electronic supplementary material

Supplementary Information

Source Data

## References

[1] Rappuoli, R., Pizza, M., Del Giudice, G. & De Gregorio, E. Vaccines, new opportunities for a new society. *Proc Natl Acad Sci USA*, 10.1073/pnas.1402981111 (2014). 10.1073/pnas.1402981111 PMC4151714 25136130

[2] UNODC. World Drug Report 2016 (2016).

[3] CDC. *Wide-ranging OnLine Data for Epidemiologic Research (WONDER)*, http://wonder.cdc.gov (2016).

[4] Volkow, N. D. & Collins, F. S. The Role of Science in Addressing the Opioid Crisis. *N Engl J Med*, 10.1056/NEJMsr1706626 (2017). 10.1056/NEJMsr1706626 28564549

[5] Pravetoni M (2016). Biologics to treat substance use disorders: Current status and new directions. Hum Vaccin Immunother.

[6] Ohia-Nwoko O, Kosten TA, Haile CN (2016). Animal Models and the Development of Vaccines to Treat Substance Use Disorders. Int Rev Neurobiol.

[7] Kosten T, Domingo C, Orson F, Kinsey B (2014). Vaccines against stimulants: cocaine and MA. Br J Clin Pharmacol.

[8] Brimijoin S, Shen X, Orson F, Kosten T (2013). Prospects, promise and problems on the road to effective vaccines and related therapies for substance abuse. Expert Rev Vaccines.

[9] Skolnick P (2015). Biologic Approaches to Treat Substance-Use Disorders. Trends Pharmacol Sci.

[10] Bremer PT, Janda KD (2017). Conjugate Vaccine Immunotherapy for Substance Use Disorder. Pharmacol Rev.

[11] Pentel PR, LeSage MG (2014). New directions in nicotine vaccine design and use. Adv Pharmacol.

[12] Diavatopoulos, D. A. & Edwards, K. M. What Is Wrong with Pertussis Vaccine Immunity? Why Immunological Memory to Pertussis Is Failing. *Cold Spring Harb Perspect Biol*, 10.1101/cshperspect.a029553 (2017). 10.1101/cshperspect.a029553 PMC5710107 28289059

[13] Coffman RL, Sher A, Seder RA (2010). Vaccine adjuvants: putting innate immunity to work. Immunity.

[14] Di Pasquale A, Preiss S, Tavares Da Silva F, Garcon N (2015). Vaccine Adjuvants: from 1920 to 2015 and Beyond. Vaccines (Basel).

[15] Pravetoni, M. *et al*. An oxycodone conjugate vaccine elicits oxycodone-specific antibodies that reduce oxycodone distribution to brain and hot-plate analgesia. *J Pharmacol Exp Ther*, 10.1124/jpet.111.189506 (2012). 10.1124/jpet.111.189506 PMC3310692 22262924

[16] Pravetoni M (2013). Reduced antinociception of opioids in rats and mice by vaccination with immunogens containing oxycodone and hydrocodone haptens. J Med Chem.

[17] Pravetoni M (2014). Effects of an oxycodone conjugate vaccine on oxycodone self-administration and oxycodone-induced brain gene expression in rats. PLoS One.

[18] Pravetoni M (2012). Co-administration of morphine and oxycodone vaccines reduces the distribution of 6-monoacetylmorphine and oxycodone to brain in rats. Vaccine.

[19] Pravetoni M (2014). Effect of currently approved carriers and adjuvants on the pre-clinical efficacy of a conjugate vaccine against oxycodone in mice and rats. PLoS One.

[20] Raleigh MD (2017). Safety and efficacy of an oxycodone vaccine: Addressing some of the unique considerations posed by opioid abuse. PLoS One.

[21] McHeyzer-Williams M, Okitsu S, Wang N, McHeyzer-Williams L (2012). Molecular programming of B cell memory. Nat Rev Immunol.

[22] Victora GD, Nussenzweig MC (2012). Germinal centers. Annu Rev Immunol.

[23] Laudenbach M (2015). The frequency of naive and early-activated hapten-specific B cell subsets dictates the efficacy of a therapeutic vaccine against prescription opioid abuse. J Immunol.

[24] Laudenbach M, Tucker AM, Runyon SP, Carroll FI, Pravetoni M (2015). The frequency of early-activated hapten-specific B cell subsets predicts the efficacy of vaccines for nicotine dependence. Vaccine.

[25] Taylor JJ, Laudenbach M, Tucker AM, Jenkins MK, Pravetoni M (2014). Hapten-specific naive B cells are biomarkers of vaccine efficacy against drugs of abuse. J Immunol Methods.

[26] Ballesteros-Tato A (2012). Interleukin-2 inhibits germinal center formation by limiting T follicular helper cell differentiation. Immunity.

[27] McDonald PW (2016). IL-7 signalling represses Bcl-6 and the TFH gene program. Nat Commun.

[28] Crotty S (2015). A brief history of T cell help to B cells. Nat Rev Immunol.

[29] Kamphorst AO, Araki K, Ahmed R (2015). Beyond adjuvants: immunomodulation strategies to enhance T cell immunity. Vaccine.

[30] Ranasinghe C, Trivedi S, Wijesundara DK, Jackson RJ (2014). IL-4 and IL-13 receptors: Roles in immunity and powerful vaccine adjuvants. Cytokine Growth Factor Rev.

[31] Jackson RJ, Worley M, Trivedi S, Ranasinghe C (2014). Novel HIV IL-4R antagonist vaccine strategy can induce both high avidity CD8 T and B cell immunity with greater protective efficacy. Vaccine.

[32] Wu LC, Scheerens H (2014). Targeting IgE production in mice and humans. Curr Opin Immunol.

[33] Berkowitz JL (2014). Safety, efficacy, and pharmacokinetics/pharmacodynamics of daclizumab (anti-CD25) in patients with adult T-cell leukemia/lymphoma. Clin Immunol.

[34] Backonja M (2016). Intravenous abuse potential study of oxycodone alone or in combination with naltrexone in nondependent recreational opioid users. Am J Drug Alcohol Abuse.

[35] Harris SC (2014). Abuse potential, pharmacokinetics, pharmacodynamics, and safety of intranasally administered crushed oxycodone HCl abuse-deterrent controlled-release tablets in recreational opioid users. J Clin Pharmacol.

[36] Jones JD, Vosburg SK, Manubay JM, Comer SD (2011). Oxycodone abuse in New York City: characteristics of intravenous and intranasal users. Am J Addict.

[37] Leow KP, Smith MT, Watt JA, Williams BE, Cramond T (1992). Comparative oxycodone pharmacokinetics in humans after intravenous, oral, and rectal administration. Ther Drug Monit.

[38] Leow KP, Smith MT, Williams B, Cramond T (1992). Single-dose and steady-state pharmacokinetics and pharmacodynamics of oxycodone in patients with cancer. Clin Pharmacol Ther.

[39] Poyhia R, Olkkola KT, Seppala T, Kalso E (1991). The pharmacokinetics of oxycodone after intravenous injection in adults. Br J Clin Pharmacol.

[40] Poyhia R, Seppala T, Olkkola KT, Kalso E (1992). The pharmacokinetics and metabolism of oxycodone after intramuscular and oral administration to healthy subjects. Br J Clin Pharmacol.

[41] Boyman O, Kovar M, Rubinstein MP, Surh CD, Sprent J (2006). Selective stimulation of T cell subsets with antibody-cytokine immune complexes. Science.

[42] Finkelman FD (1993). Anti-cytokine antibodies as carrier proteins. Prolongation of *in vivo* effects of exogenous cytokines by injection of cytokine-anti-cytokine antibody complexes. J Immunol.

[43] Phelan JD, Orekov T, Finkelman FD (2008). Cutting edge: mechanism of enhancement of *in vivo* cytokine effects by anti-cytokine monoclonal antibodies. J Immunol.

[44] Verdeil G, Marquardt K, Surh CD, Sherman LA (2008). Adjuvants targeting innate and adaptive immunity synergize to enhance tumor immunotherapy. Proc Natl Acad Sci USA.

[45] Urban JF, Maliszewski CR, Madden KB, Katona IM, Finkelman FD (1995). IL-4 treatment can cure established gastrointestinal nematode infections in immunocompetent and immunodeficient mice. J Immunol.

[46] Fanoe S, Jensen GB, Sjogren P, Korsgaard MP, Grunnet M (2009). Oxycodone is associated with dose-dependent QTc prolongation in patients and low-affinity inhibiting of hERG activity *in vitro*. Br J Clin Pharmacol.

[47] Kerdine S, Lebrec H, Bertoglio J, Pallardy M (1996). Interleukin-4 plays a dominant role in Th1- or Th2-like responses during the primary immune response to the hapten penicillin. Mol Immunol.

[48] Kerdine S, Pallardy M, Lamanetre S, Bertoglio J, Lebrec H (1996). Interleukin-10 and interleukin-4 have similar effects on hapten-specific primary antibody responses to penicillin *in vivo*. Eur J Immunol.

[49] Mathers AR, Cuff CF (2004). Role of interleukin-4 (IL-4) and IL-10 in serum immunoglobulin G antibody responses following mucosal or systemic reovirus infection. J Virol.

[50] Bremer PT, Janda KD (2012). Investigating the effects of a hydrolytically stable hapten and a Th1 adjuvant on heroin vaccine performance. J Med Chem.

[51] Bremer PT, Schlosburg JE, Lively JM, Janda KD (2014). Injection route and TLR9 agonist addition significantly impact heroin vaccine efficacy. Mol Pharm.

[52] McCluskie MJ (2013). Enhancing immunogenicity of a 3’aminomethylnicotine-DT-conjugate anti-nicotine vaccine with CpG adjuvant in mice and non-human primates. Int Immunopharmacol.

[53] Zheng H (2015). Negatively Charged Carbon Nanohorn Supported Cationic Liposome Nanoparticles: A Novel Delivery Vehicle for Anti-Nicotine Vaccine. J Biomed Nanotechnol.

[54] Hu Y (2016). The next-generation nicotine vaccine: a novel and potent hybrid nanoparticle-based nicotine vaccine. Biomaterials.

[55] Bruhns P (2012). Properties of mouse and human IgG receptors and their contribution to disease models. Blood.

[56] Fritz JM (2014). Depletion of tumor-associated macrophages slows the growth of chemically induced mouse lung adenocarcinomas. Front Immunol.

[57] Baumjohann D (2013). Persistent antigen and germinal center B cells sustain T follicular helper cell responses and phenotype. Immunity.

[58] Kimishima A, Wenthur CJ, Zhou B, Janda KD (2017). An Advance in Prescription Opioid Vaccines: Overdose Mortality Reduction and Extraordinary Alteration of Drug Half-Life. ACS Chem Biol.

[59] Moon JJ (2007). Naive CD4(+) T cell frequency varies for different epitopes and predicts repertoire diversity and response magnitude. Immunity.

[60] Tubo NJ (2013). Single Naive CD4(+) T Cells from a Diverse Repertoire Produce Different Effector Cell Types during Infection. Cell.

