# Supplementary material for: Blocking interleukin-4 enhances efficacy of vaccines for treatment of opioid abuse and prevention of opioid overdose
Source: Sci Rep. 2018 Apr 3;8:5508. doi: 10.1038/s41598-018-23777-6 (PMC5882912; doi:10.1038/s41598-018-23777-6)
Supplement: Supplementary file 1 — Supplementary Information [file 41598_2018_23777_MOESM1_ESM.pdf]

Blocking interleukin-4 enhances efficacy of vaccines for treatment of opioid abuse and prevention of opioid overdose

Megan Laudenbach, Federico Baruffaldi, Christine Robinson, Philipp Carter, Davis Seelig, Carly Baehr, and Marco Pravetoni

Supplementary Information

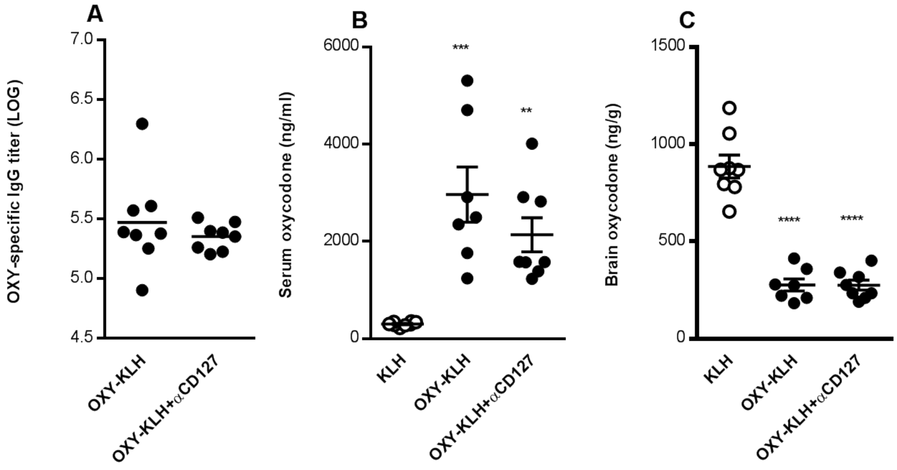

Supplemental Figure S1. IL-7 receptor blockage does not enhance OXY-KLH efficacy. Male BALB/c mice were immunized s.c. on days 0, 14, and 28, and challenged with 2.25 mg/kg s.c. oxycodone a week after the 3rd immunization. Mice received unconjugated KLH, OXY-KLH, or OXY-KLH plus an anti-IL-7α receptor mAb (αCD127, 1.0 mg per mouse, intraperitoneally). The OXY-KLH and KLH were adsorbed on alum adjuvant prior to administration. The mAb was administered 2 days prior and 1 day after the 1st immunization. A) oxycodone-specific IgG antibody titers, and effect of immunization on oxycodone distribution to B) serum and C) brain. Data are mean±SEM. Data shown are from one experiment (n=8). One-way ANOVA paired with Tukey's multiple comparisons. $^{**}p<0.01$, $^{***}p<0.001$, $^{****}p<0.0001$ compared to KLH control.

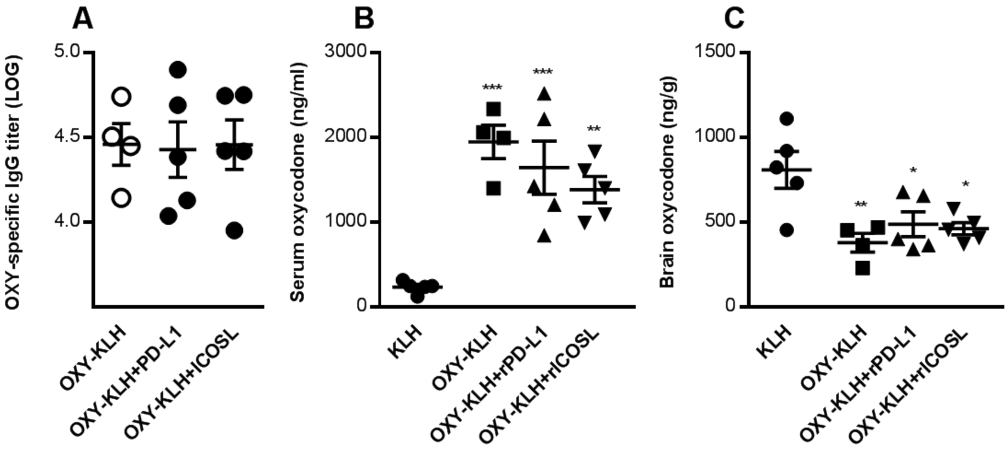

Supplemental Figure S2. **Co-stimulatory molecules do not enhance OXY-KLH efficacy.** Male BALB/c mice were immunized s.c. on days 0, 14, and 28, and challenged with 5 mg/kg s.c. oxycodone a week after the 3rd immunization. Mice received KLH, OXY-KLH, or OXY-KLH plus either recombinant Programmable Death Ligand 1 (rPD-L1) or recombinant ICOS ligand (rICOSL). The OXY-KLH and the unconjugated KLH were adsorbed on alum adjuvant prior to administration. PD-L1 and ICOSL were co-administered s.c. with vaccines. A) oxycodone-specific IgG antibody titers, effect of immunization on oxycodone distribution to B) serum and C) brain. Data are mean±SEM. Data shown are from one experiment (n=5). One-way ANOVA paired with Tukey's multiple comparisons. *p<0.05, **p<0.01, ***p<0.001, ****p<0.0001 compared to KLH control.

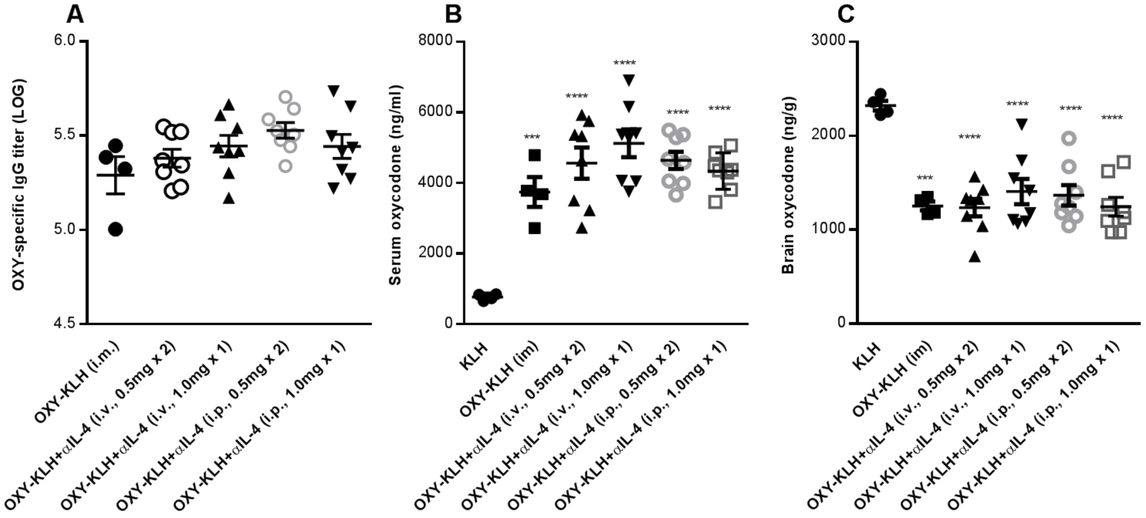

Supplemental Figure S3. Co-administration of OXY-KLH plus αIL-4 mAb is effective across clinically-relevant immunization regimens. Male BALB/c mice were immunized i.m. with OXY-KLH or KLH on days 0, 14 and 28, and challenged with 5.0 mg/kg oxycodone a week after the third immunization. Mice received KLH (n=4), OXY-KLH (n=4), or OXY-KLH plus the αIL-4 mAb (n=8). As labeled, αIL-4 mAb was administered either i.p. or i.v., either as 1 dose (1.0 mg) or 2 doses (0.5mg each). Data are from one experiment and expressed as mean±SEM. One-way ANOVA paired with Tukey's multiple comparisons. ***$p<0.001$, ****$p<0.0001$ compared to KLH.

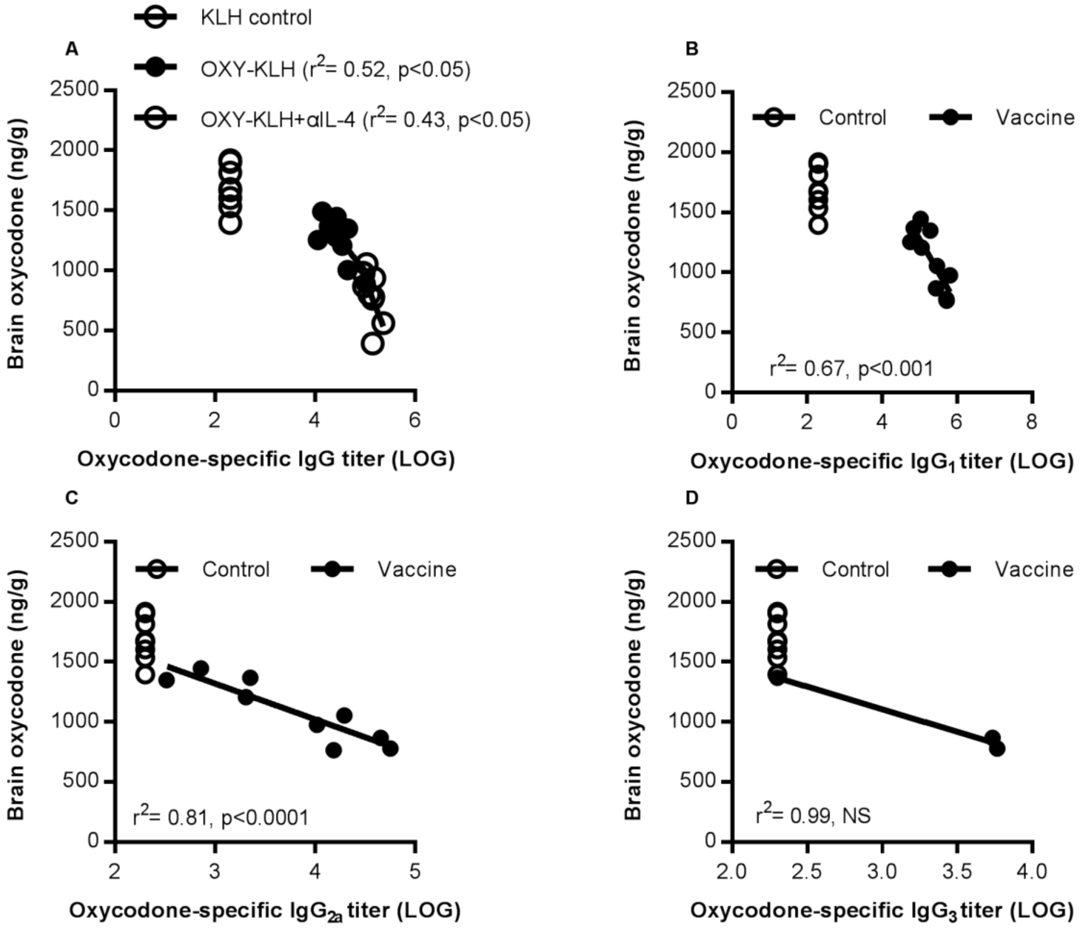

Supplemental Figure S4. Increased oxycodone-specific serum IgG, IgG_1, and IgG_{2a} antibody titers correlated with reduction in brain oxycodone. A-D) Analysis was performed on individual mice challenged with 5mg/kg oxycodone and reported in Figure 1E-G. A) Linear regression of oxycodone-specific serum IgG titers and brain concentration in mice treated with either OXY-KLH or OXY-KLH plus αIL-4 mAb (n=10 mice each group). The individual brain oxycodone concentrations in the KLH control group are provided as comparison (n=10). B-D) Linear regression of oxycodone-specific serum IgG_1, IgG_{2a}, and IgG_3 titers and brain concentration in immunized mice. The vaccine treatment group includes mice immunized with OXY-KLH (n=5) and OXY-KLH plus αIL-4 mAb (n=5). D) Most individual mice in this cohort had little to no detectable IgG_3 titers.

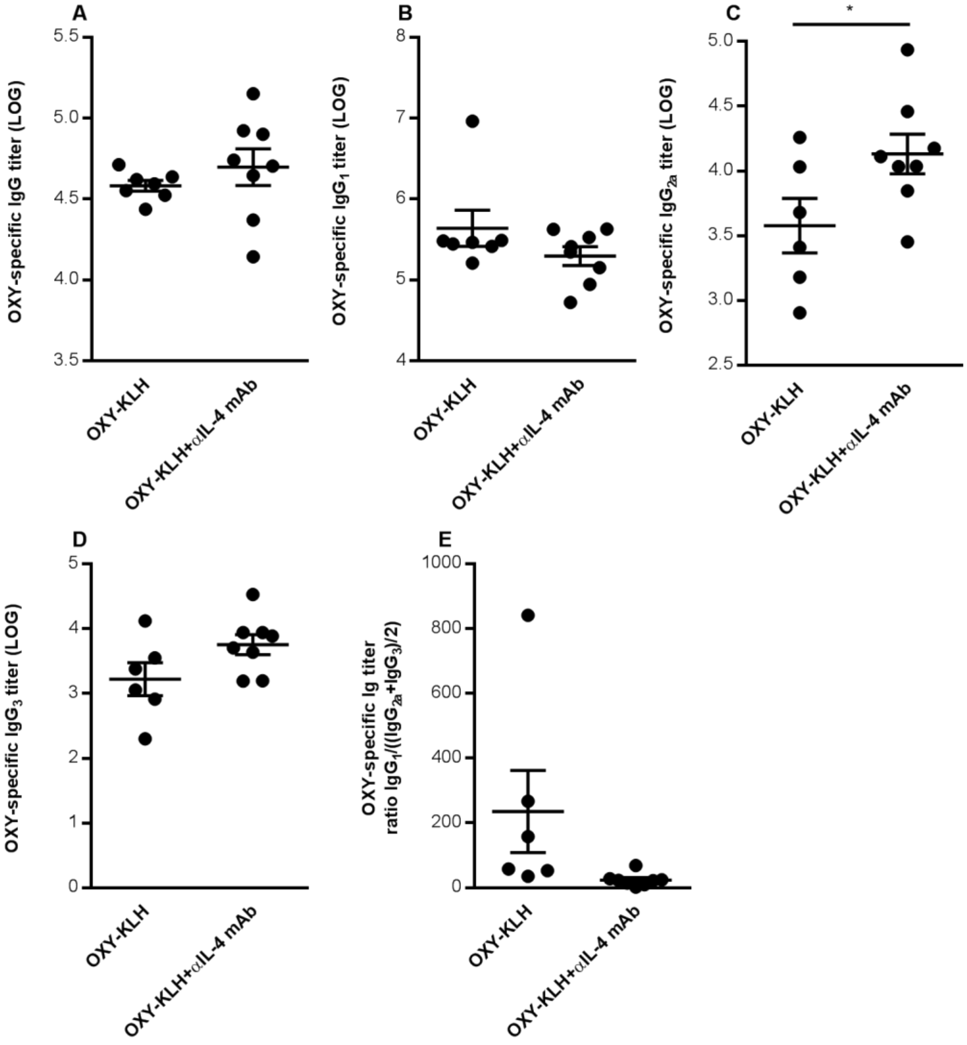

Supplemental Figure S5. Co-administration of OXY-KLH and αIL-4 mAb increases anti-oxycodone serum IgG_{2a} antibodies in C57Bl/6 mice. Male C57Bl/6 mice were immunized s.c. with OXY-KLH or OXY-KLH plus αIL-4 mAb i.p., and anti-oxycodone serum IgG antibodies were analyzed for IgG_1, IgG_{2a}, and IgG_3 subclass titers: A) total oxycodone-specific IgG titers, B) IgG_1, C) IgG_{2a}, D) IgG_3, and E) $IgG_1/((IgG_{2a} + IgG_3)/2)$. Data are from one experiment (n=8) and expressed as mean±SEM. Unpaired two-tailed t-test. *$p<0.05$.

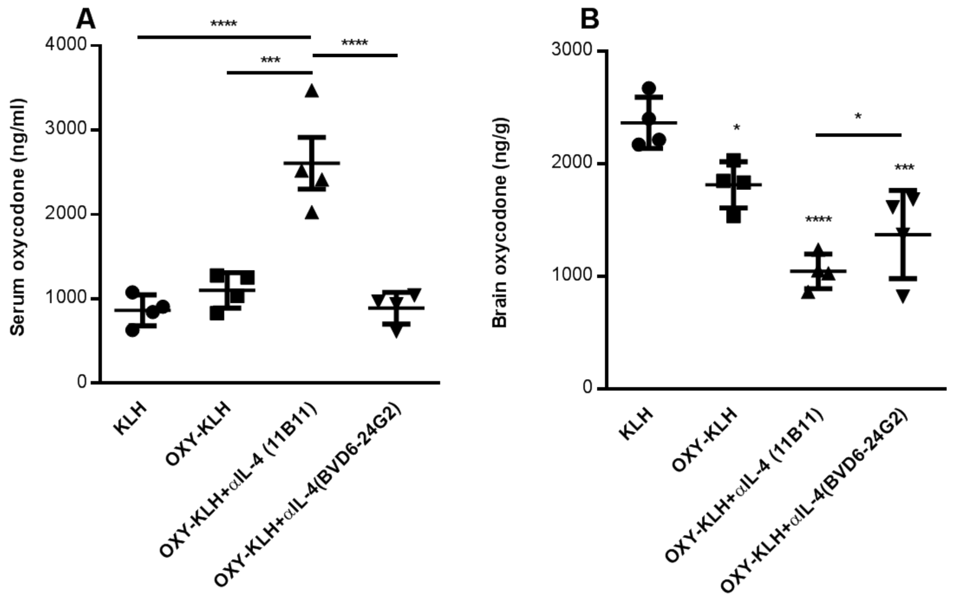

Supplemental Figure S6. The OXY-KLH efficacy is enhanced by neutralizing αIL-4 mAb but not by non-neutralizing αIL-4 mAb. Male BALB/c mice were immunized i.m. with KLH or OXY-KLH on days 0, 14 and 28, and challenged with 5.0 mg/kg oxycodone a week after the third immunization. Mice received KLH (n=4), OXY-KLH (n=4), and OXY-KLH plus either neutralizing αIL-4 mAb (11B11, n=4) or non-neutralizing αIL-4 mAb (BVD6-24G2, n=4). The αIL-4 mAb was administered i.p. as 2 doses (0.5mg each). Data are from one experiment and expressed as mean±SEM. One-way ANOVA paired with Tukey's multiple comparisons. *p<0.05, ***p<0.001, and ****p<0.0001 compared to KLH or brackets to indicate significance between groups.

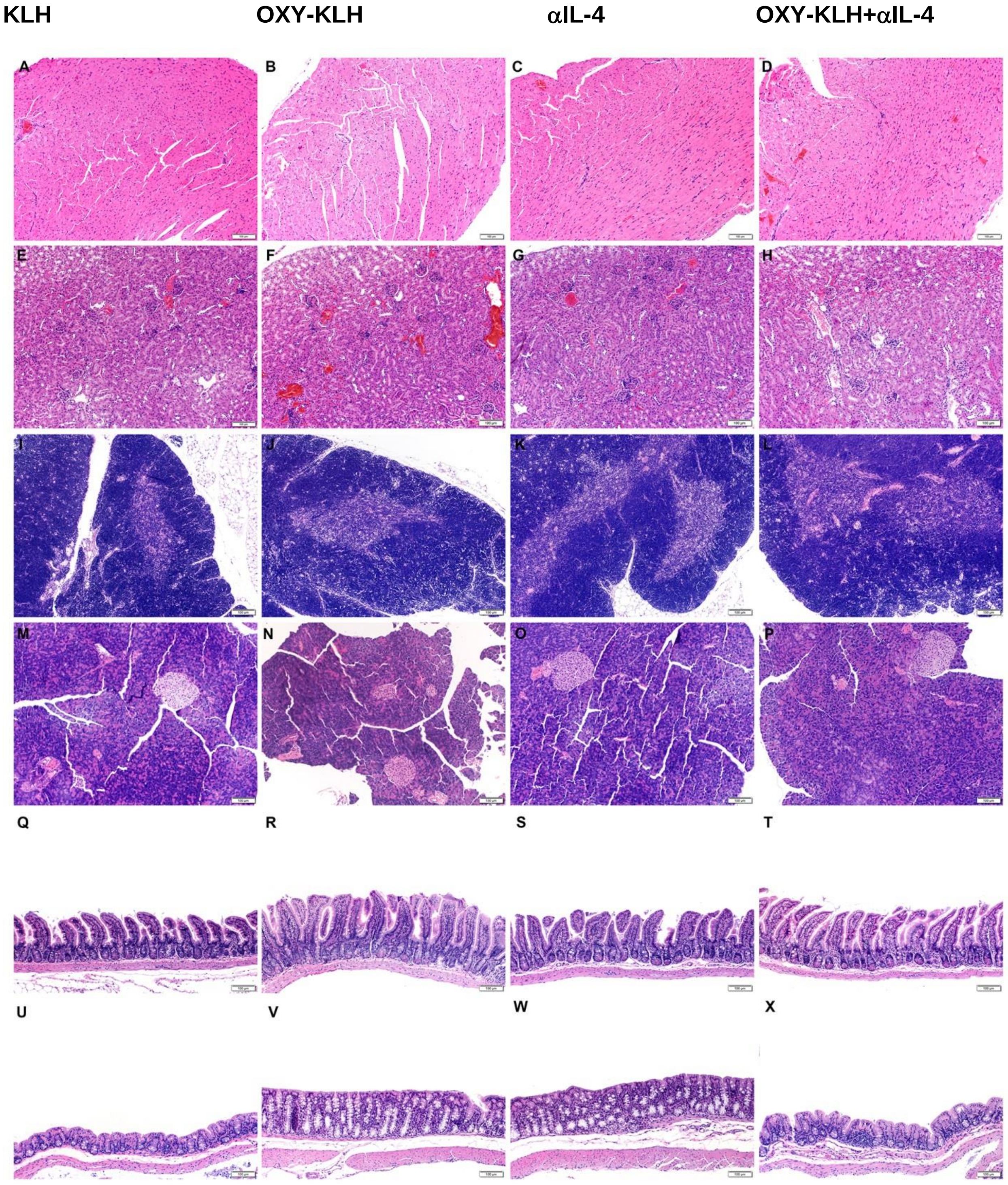

Supplemental Figure S7. Co-administration of OXY-KLH plus αIL-4 mAb is safe. No significant pathology was identified in A-D) heart, E-H) kidney, I-L) thymus, M-P) pancreas, Q-T) small intestine, and U-X) large intestine in mice immunized with KLH (s.c.), OXY-KLH (s.c.), αIL-4 (i.p.), or OXY-KLH (s.c.) plus αIL-4 (i.p.).

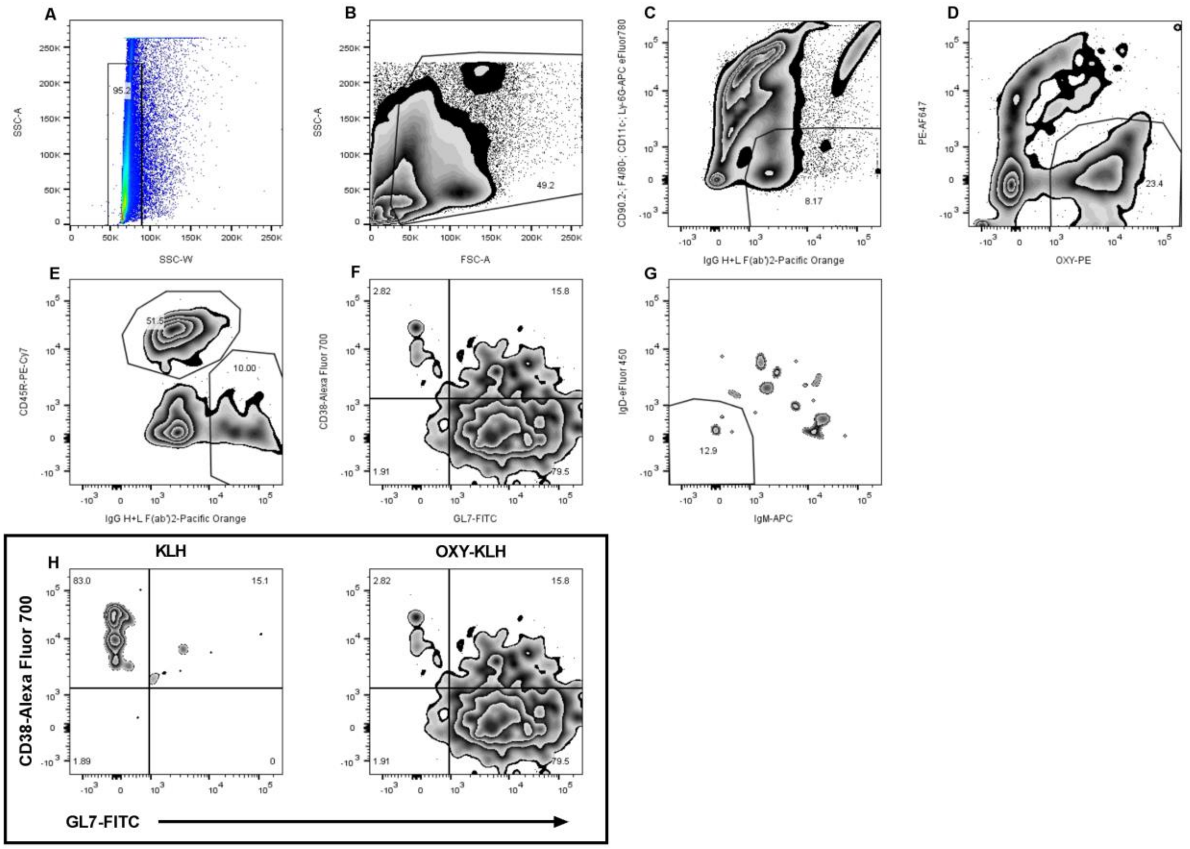

Supplemental Figure S8. Gating strategy for flow cytometric analysis of hapten-specific B cells and characterization of hapten-specific germinal center (GC) B cells from mouse lymphoid organs 7 d post-vaccination with OXY-KLH or unconjugated KLH control. A) Singlet gating to remove any aggregated cells. B) Total lymphocytes. C) Total B cells expressing gamma immunoglobulin G heavy and light chain (IgG+ [H+L]) and excluding the following non-B cell markers: CD90.2 (T cells), Gr-1 (neutrophils), CD11.c (dendritic cells), and F4/80 (macrophages). D) OXY-specific B cells bound to OXY-Streptavidin-PE were differentiated from B cells bound to a Streptavidin-PE^{AF647} decoy (1,2). E) OXY-specific B cells were identified as either non antibody-secreting cells (non-ASC) expressing CD45R^{high} or antibody-secreting cells (ASC) expressing high levels of gamma immunoglobulin (IgG^{high}). F) OXY-specific CD45R^{high} non-ASC B cells were identified as GL7^{+} GC, CD38^{+} naïve/memory (N/M), or GL7^{+}/CD38^{+} precursor memory B cells. G) N/M B cells were identified as IgM^{high} or IgM^{low}/IgD^{low} switched immunoglobulin (swIg) cells. H) Representative comparison of CD38 and GL7 expression in mice immunized with either KLH or OXY-KLH adsorbed on alum adjuvant.

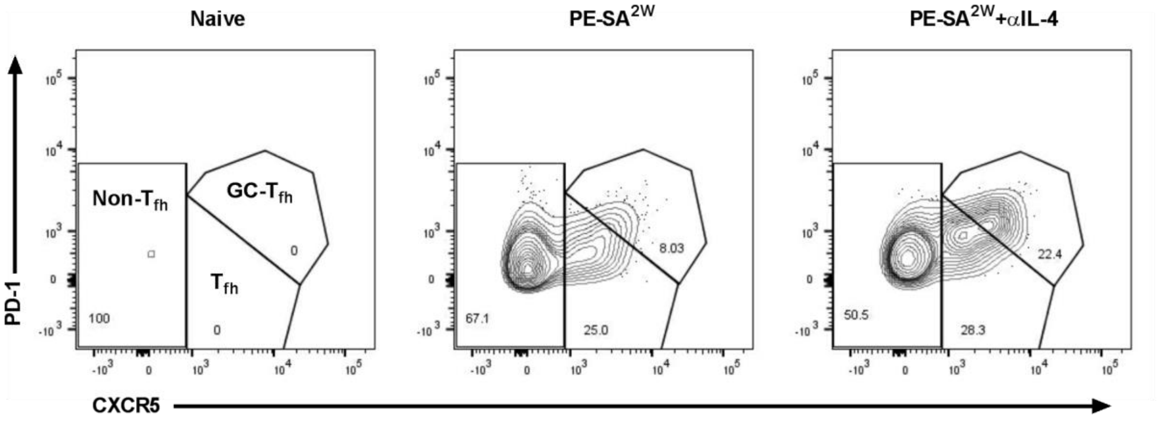

Supplemental Figure S9. Gating strategy for analysis of 2W1S:MHCII-restricted T cells in C57Bl/6 mice immunized with PE-SA^{2W} with or without αIL-4 mAb, or naïve mice as control. Analysis of 2W-specific *CD4*$^{+}$ T cells was performed as described (1, 3, 4). Lymph nodes and spleens are processed to single-cell suspensions. Samples are incubated with 2W1S:I-A^{b} tetramers conjugated to PE and APC, and BV421 anti-CXCR5 (3). Samples are then enriched with anti-PE and anti-APC magnetic beads and magnetic columns (3). After magnetic enrichment, samples are labeled with PE-Cy7 labeled CD45R, CD11b/c, CD11c, F4/80 antibodies to identify non-T cell populations, and then with T cell markers: PE anti-CD4, BV510 anti-CD8a, APC-eFluor780 anti-CD4, PerCP-eFluor710 anti-CD90.2, FITC antiCD279 (PD-1), and AF700 anti-CD44. Flow cytometric analysis: a) singlets through standard FSC-W and FSC-A, b) T cells are CD90.2^{high} not expressing non-T cell markers, which are then classified as CD8^{high} T cells or CD4^{high} T cells, c) Within the CD4^{high} T cell subset, 2W1S-specific CD4^{+} T cells are identified as PE^{high} and APC^{high}, and d) 2W1S-specific CD4^{+} T cells are classified as CXCR5^{neg} non-Tfh, CXCR5^{mid} PD-1^{neg} Tfh, and CXCR5^{high} PD-1^{+} GC-Tfh (4).

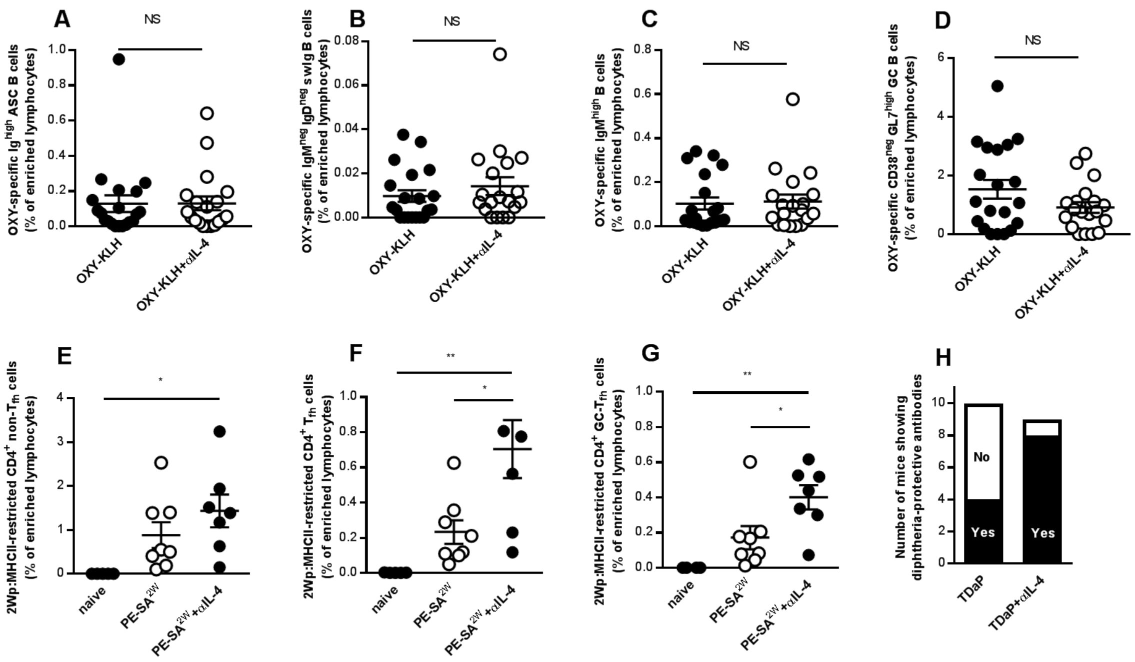

Supplemental Figure S10. A-G) Data from Figure 5A-F expressed as percentages of lymphocytes in enriched samples from individual mice. Analysis of the OXY-specific B cell population: A) plasma cells or antibody-secreting cells (ACS), B) switched immunoglobulin (swIg), C) IgM^{high} naïve and memory, and D) germinal center (GC). Analysis of the 2Wp:MHCII-restricted $CD4^{+}$ T cell population: E) non-Tfh, F) Tfh and G) GC-Tfh. *p<0.05, **p<0.01. H) Number of mice that showed detectable (IC_{50}>10) protective antibody titers against diphtheria toxin. Chi-square analysis, $\chi 2$, df= 4.866, 1, z=2.206, p<0.05, two tailed.

Supplemental table S1. Effect of combining immunomodulators with vaccines on baseline nociception.

Formulation	Sample size	Baseline nociception (s)	Body weight (g)
KLH	n=49	17.04±1.03	27.20±0.26
OXY-KLH	n=48	13.93±0.57*	26.80±0.29
OXY-KLH + αIL-4	n=38	15.22±0.73	27.13±0.22
OXY-KLH + αCD25	n=20	12.38±1.03**	26.02±0.25*

Data are from mice immunized with KLH (s.c.), OXY-KLH (s.c.), OXY-KLH (s.c.) plus either αIL-4 or αCD25 mAb (i.p.) from experiments presented in Figures 1 and 2. Nociception is expressed as delay to hind paw lifts on a hotplate set at 54°C. Data are expressed as mean±SEM. One-way ANOVA paired with Tukey's multiple comparisons test. *$p<0.05$ and **$p<0.01$ from KLH control.

Supplemental table S2. Combining immunomodulators with vaccines: blood biochemistry.

Treatment	Protein, total (g/dL)	Bilirubin, total (mg/dL)	ALT (U/L)	AST (U/L)	Urea nitrogen (mg/dL)	Cholesterol (mg/dL)	Body weight (g)
KLH	5.7 ± 0.32	0.2 ± 0.10	71.6 ± 3.2	626 ± 24.7	20 ± 1.5	161 ± 10.4	26.5 ± 0.39
OXY-KLH	5.4 ± 0.17	0.3 ± 0.11	74 ± 4.7	709 ± 51.6	20 ± 1.0	154 ± 11.0	27.4 ± 0.75
αIL-4 mAb	5.8 ± 0.06	0.46 ± 0.03	104 ± 9.5	605 ± 253	27 ± 2.8	163 ± 0.3	27.7 ± 0.72
OXY-KLH + αIL-4 mAb	5.5 ± 0.26	0.27 ± 0.09	101 ± 32.3	840 ± 286	24 ± 1.0	158 ± 13.0	27.1 ± 0.24
Reference Values*	4.2 - 5.8	0 - 0.5	41 - 131	55 - 32	7 - 26	111 - 246	

Blood biochemistry analysis in BALB/c mice immunized with KLH (s.c.), OXY-KLH (s.c.), αIL-4 mAb (i.p.), and OXY-KLH (s.c.) plus αIL-4 mAb (i.p.). Reported values for protein, bilirubin, ALT (alanine aminotransferase), AST (aspartate aminotransferase), urea nitrogen and total cholesterol. Data are from one experiment and reported as mean±SEM (n=3/group). One way ANOVA paired with Tukey's multiple comparisons test. *Reference values for BALB/c mice as reported from the Jackson Laboratory.

Supplemental table S3. List of reagents and antibodies for flow cytometry analysis.

Reagent	Catalog #	Manufacturer
**B cell analysis**		
OXY(Gly)$_4$-peptide-biotin-streptavidin-PE	--	Custom, Pravetoni
NH_2-peptide-biotin-streptavidin-PE^{AF647}	--	Custom, Pravetoni
FITC rat-anti-mouse T and B cell Activation Antigen (clone GL7)	55366	BD Pharmingen
CD45R (B220) PE-Cyanine7 (Clone: RA3-6B2) (formerly eBioscience)	25-0452-82	ThermoFisher Scientific
CD38 Alexa Fluor® 700 (Clone: 90)	56-0381-82	ThermoFisher Scientific
IgM APC (Clone: II/41)	17-5790-82	ThermoFisher Scientific
CD11c APC-eFluor® 780 (Clone: N418)	47-0114-82	ThermoFisher Scientific
CD90.2 (Thy-1.2) APC-eFluor® 780 (Clone: 53-2.1)	47-0902-82	ThermoFisher Scientific
F4/80 Antigen APC-eFluor® 780 (Clone: BM8)	47-4801-82	ThermoFisher Scientific
Ly-6G (Gr-1) APC-eFluor® 780 (Clone: RB6-8C5)	47-5931-82	ThermoFisher Scientific
IgD eFluor® 450 (Clone: 11-26c)	48-5993-82	ThermoFisher Scientific
IgG H+L F(ab')2 Pacific Orange	P31585	Invitrogen (Life Technologies)
**B cell compensation staining**		
CD4 FITC (Clone: GK1.5)	11-0041-85	ThermoFisher Scientific
CD4 PE (Clone: GK1.5)	12-0041-83	ThermoFisher Scientific
CD4 PE-Cyanine5 (Clone: GK1.5)	15-0041-83	ThermoFisher Scientific
**T cell analysis**		
2Wp:MCHII-PE and –APC	--	Custom, Marc Jenkins
CD44 AlexaFluor700 clone IM7	56-0441-82	ThermoFisher Scientific
CD45R (B220) PE-Cy7 clone RA3-6B2	25-0452-82	ThermoFisher Scientific
CD11b PE-Cy7 clone M1/70	552850	BD biosciences
CD11c PE-Cy7 clone N418	25-0114-82	ThermoFisher Scientific
CD279 (PD-1) FITC clone J43	11-9985-82	ThermoFisher Scientific
CD4 APC clone GK1.5	17-0041-82	ThermoFisher Scientific
CD4 APC-eFluor780 clone RM4-5	47-0042-82	ThermoFisher Scientific
CD8a BV510 clone 53-6.7	563068	BD biosciences
CD90.2 (Thy-1.2) PerCP-eFluor710 clone 30-H12	46-0903-82	ThermoFisher Scientific
CXCR5 BV421 clone 2G8	562889	BD biosciences
F4/80 PE-Cy7 clone BM8	25-4801-82	ThermoFisher Scientific

Supplemental figure references

1. Laudenbach M, Baruffaldi F, Vervacke JS et al. The frequency of naive and early-activated hapten-specific B cell subsets dictates the efficacy of a therapeutic vaccine against prescription opioid abuse. J Immunol 2015;194:5926-36.
2. Taylor JJ, Laudenbach M, Tucker AM, Jenkins MK, Pravetoni M. Hapten-specific naive B cells are biomarkers of vaccine efficacy against drugs of abuse. J Immunol Methods 2014;405:74-86.
3. Tubo NJ, Pagan AJ, Taylor JJ et al. Single Naive CD4(+) T Cells from a Diverse Repertoire Produce Different Effector Cell Types during Infection. Cell 2013;153:785-96.
4. Yang JA, Tubo NJ, Gearhart MD, Bardwell VJ, Jenkins MK. Cutting edge: Bcl6-interacting corepressor contributes to germinal center T follicular helper cell formation and B cell helper function. J Immunol. 2015 Jun 15;194(12):5604-8.
